# Social Capital and Health: A Review of Prospective Multilevel Studies

**DOI:** 10.2188/jea.JE20110128

**Published:** 2012-05-05

**Authors:** Hiroshi Murayama, Yoshinori Fujiwara, Ichiro Kawachi

**Affiliations:** 1 Research Team for Social Participation and Community Health, Tokyo Metropolitan Institute of Gerontology, Tokyo, Japan; 2 Department of Society, Human Development, and Health, Harvard School of Public Health, Boston, USA

**Keywords:** health, literature review, multilevel analysis, prospective study, social capital

## Abstract

**Background:**

This article presents an overview of the concept of social capital, reviews prospective multilevel analytic studies of the association between social capital and health, and discusses intervention strategies that enhance social capital.

**Methods:**

We conducted a systematic search of published peer-reviewed literature on the PubMed database and categorized studies according to health outcome.

**Results:**

We identified 13 articles that satisfied the inclusion criteria for the review. In general, both individual social capital and area/workplace social capital had positive effects on health outcomes, regardless of study design, setting, follow-up period, or type of health outcome. Prospective studies that used a multilevel approach were mainly conducted in Western countries. Although we identified some cross-sectional multilevel studies that were conducted in Asian countries, including Japan, no prospective studies have been conducted in Asia.

**Conclusions:**

Prospective evidence from multilevel analytic studies of the effect of social capital on health is very limited at present. If epidemiologic findings on the association between social capital and health are to be put to practical use, we must gather additional evidence and explore the feasibility of interventions that build social capital as a means of promoting health.

## INTRODUCTION

The effectiveness and efficiency of community-based health promotion programs vary depending on their context and location, even when the programs have a similar design. Such variation may be due to differences in the background characteristics of the settings in which the interventions are conducted. One such characteristic is “social capital,” a concept that has been used in recent years to explain health disparities. Social capital might provide a theoretical basis for assessing the impact that community-based health promotion programs have on the broader health and life of a community.^1^ In this article, we provide an overview of the concept of social capital, discuss previous empirical research, and identify intervention strategies that enhance social capital.

### Definition of social capital

**Figure 1.**
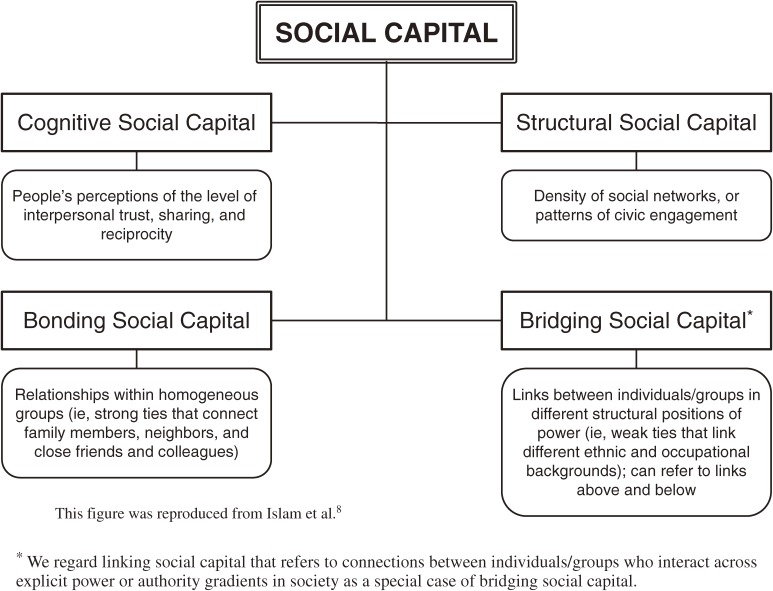
Conceptual arrangement of social capital

Coleman defined social capital as “not a single entity, but a variety of different entities having two characteristics in common: They all consist of some aspect of social structure, and they facilitate certain actions of individuals who are within the structure”.^2^ In addition, according to Putnam, social capital refers to “features of social organization, such as trust, norms and networks, that can improve the efficacy of society by facilitating coordinated actions”.^3^ The existing literature highlights 2 distinct concepts of social capital.^4^ The first is that social capital represents the resources available to members of tightly knit communities. This interpretation could be described as the “social cohesion” definition. Social cohesion tends to emphasize social capital as a group attribute and analyze it as a contextual influence on individual health. In contrast, the “network” theory of social capital defines the concept in terms of resources that are embedded within an individual’s social networks, that is, it is regarded as a property of individuals.^5^ To date, the most common approach to defining social capital in research on population health has been the social cohesion perspective, ie, social capital conceptualized as an attribute of a collective (eg, neighborhoods, workplaces, schools). Social capital can be broken down into a number of forms and dimensions. A common distinction in research on social capital is between structural and cognitive dimensions.^3^ The structural dimension includes externally observable aspects of social organization and is characterized by behavioral manifestations of network connections or civic engagement. The cognitive dimension reflects subjective attitudes such as trust in others and norms of reciprocity. An additional distinction has been drawn between bonding and bridging social capital.^3^ Bonding social capital refers to trusting and cooperative relations within homogeneous groups, that is, the strong ties between members of a network who are similar in terms of sociodemographic or social characteristics (eg, age, ethnicity, social class). Bridging social capital describes relations between individuals who are dissimilar with respect to social identity and power.^4^^,^^6^^,^^7^ Figure 1 shows the conceptual arrangement of social capital.

### Social capital and health

Kawachi and Berkman identified at least 8 fields of social inquiry that have examined the links between social capital and diverse outcomes, including: (1) families and youth behavior problems, (2) schooling and education, (3) community life, (4) work and organizations, (5) democracy and governance, (6) economic development, (7) criminology, and (8) public health.^9^

There is considerable evidence of an association between social capital and various indicators of health. Kawachi et al reported ecologic associations between social capital and mortality in 39 US states.^10^ Their research provided strong ecologic evidence of a relationship between state-level social mistrust and mortality rates and between state per capita group membership and mortality rates. In addition, individual-level evidence has also been presented in numerous studies (summarized in Kawachi et al^11^).

Although both ecologic and individual-level studies of social capital have yielded useful insights, a proper examination of social capital as a collective (and contextual) influence on health requires multilevel analysis.^12^ In social cohesion theory, social capital is a contextual concept. Macinko and Starfield identified 4 analytic levels in the association between social capital and health^13^: the macro level (countries, states, regions, and local municipalities), meso level (neighborhoods and blocks), micro level (social networks and social participants), and individual psychological level (trust and norm). Some researchers have studied the relationship between social capital and health at the macro and meso levels (ie, ecologic studies), while others have done so at the micro and psychological levels (individual-level studies). To examine the influence of the contextual effect of social capital on individual health outcomes over and above the individual effect, a multilevel approach needs to be adopted in studies of social capital and health. In addition, a multilevel approach enables detailed examination of cross-level interactions, such as those between community social cohesion and individual characteristics.

A multilevel framework simultaneously examines groups (eg, area, neighborhood) and the individuals nested within them and offers a comprehensive framework for understanding the ways in which places affect people (contextual effect) or, alternatively, how people can affect the groups or places to which they belong (compositional effect). Variability can be examined at both the group level and the individual level, and the role of group-level and individual-level constructs can be investigated to explain variation in outcomes among individuals and groups. Adopting a multilevel framework implies that variations in health outcomes are determined by both individual risk and protective factors, as well as by community risk and resilience factors. Thus, interventions to mitigate adverse health outcomes can be offered at both the individual and community level.^12^ Multilevel analysis can be used for 2 purposes: (1) to examine between-group and within-group variability in outcomes and the degree to which between-group variability is accounted for by group-level and individual-level variables and (2) to estimate associations between group characteristics and individual-level outcomes after adjustment for individual-level confounders.^14^

Kawachi and Berkman discussed the mechanisms by which social capital exerts a contextual effect on individual health. They identified 4 plausible pathways: diffusion of knowledge on health promotion, maintenance of healthy behavioral norms through informal social control, promotion of access to local services and amenities, and psychological processes that provide affective support and mutual respect.^9^

The contextual unit used has varied across studies. Previous studies have adopted widely varying spatial scales as their contextual unit of analysis, ranging from whole countries,^15^ states or prefectures within countries,^10^^,^^16^^–^^18^ local municipalities,^19^^–^^21^ postal code areas,^22^^–^^24^ small-area neighborhoods (eg, enumeration districts, administrative districts),^25^^–^^27^ companies,^28^^,^^29^ functional work units within workplaces,^30^^–^^32^ and schools.^33^ However, these definitions of contextual level suggest an important problem in multilevel analysis. Failure to identify the correct entity at the contextual level can result in a lack of association even when a contextual level association is actually present. For example, the effects of social capital on crime control were reported to vary depending on changes in the geographic range of a neighborhood.^34^ Thus, specifying the spatial scale for social capital requires sound theory.

To explain the mechanism by which social capital influences health, it is essential to establish a causal relationship between social capital and health. Impediments to causal inference include the possibility of reverse causality, ie, good health may be a determinant of social capital rather than the reverse.^35^ Identifying a causal relationship between social capital and health would contribute to the development of intervention strategies. Prospective data analysis is an established method to improve causal inference (versus cross-sectional studies). Of course, specifying the correct temporal sequence between exposure (social capital) and outcome (health) is only the first step. Additional obstacles to causal inference need to be addressed, including confounding by omitted variables at both the individual and group levels. During the course of our systematic review, we identified several prospective studies that examined the influence of social capital on health outcomes, including studies on mortality (including suicide),^36^^–^^38^ self-rated health,^39^^,^^40^ and depression.^41^^–^^44^ In general, these studies show a protective effect of social capital on adverse health outcomes. However, many of these prospective studies only examined individual-level associations between perceptions of social capital and health outcomes.

As mentioned above, a multilevel approach is an effective tool when using prospective data to accumulate robust evidence of an association between social capital and health. We reviewed prospective multilevel analytic studies to investigate the association between social capital and health.

## METHODS

We used the PubMed database to conduct a systematic search of peer-reviewed studies published up to 31 August 2011. The following keywords were used in the search: [“social capital” OR “social cohesion” OR “collective efficacy”], [“health”], [“multilevel” OR “contextual effect”], and [“prospective” OR “longitudinal” OR “cohort study”]. The keywords were combined in the searches. We mainly included studies that examined the direct contextual association between social capital and health. When the searches were completed, we first reviewed the title, keywords, and abstracts. If this initial review suggested that the study was relevant, we then reviewed the full text of the article for final selection. Articles published in languages other than English were excluded.

## RESULTS

These search strategies identified 13 articles suitable for review. The Table shows the sources and characteristics of the 13 reviewed articles, including study country and setting, year of survey, study subjects, measure of social capital, outcome variables, analytic strategy, and main findings. Most of the articles were from northern Europe (Finland and Sweden: 8 articles). The study setting was divided into 2 types: community (9 articles) and workplace (4 articles). We found several definitions of the analytic unit of contextual effect: neighborhood was defined by, for example, ZIP code area, electoral ward, administrative area,^23^^,^^24^^,^^45^^–^^49^ municipality^50^ or state,^51^ and functional work unit.^30^^–^^32^^,^^52^

**Table.** Prospective multilevel analytic studies of the association between social capital and health

Author	Country	Setting	Year of survey	Study subjects	Social capital	Outcome	Analysis	Key findings
Snelgrove et al^23^	United Kingdom	Community	Baseline: 1998–1999 Follow-up: 2003	Community-dwelling residents (*n* = 3075) in 250 postcode sectors	Social trust and civic participation at individual level and area level (aggregated).	Self-rated health	Multilevel logistic regression analysis	High individual and area social trust were inversely associated with poor self-rated health, but civic participation was not associated with individual or area levels after adjustment for sociodemographic characteristics and health-related behaviors.

Wen et al^24^	United States	Community	Baseline: 1993 (individual-level), 1994–1995 (contextual social environment), and 1999 (contextual socioeconomic status) Follow-up: until 1999	Patients newly diagnosed in 1993 with 1 of 13 serious illnesses (eg, acute myocardial infarction, congestive heart failure, central nervous system) (*n* = 12 672) in 51 ZIP code areas in Chicago	Collective efficacy (7 items; ie, mutual help, social trust), social network density (4 items), social support (4 items), local organizations and voluntary associations (the numbers of these organizations in the area) at the ZIP code area level (aggregated). (No individual-level social capital variable was used.)	All-cause mortality	Multilevel Cox proportional hazards model	Contextual collective efficacy had a protective effect on mortality, whereas community social network density was detrimental. Social support, local organizations, and voluntary associations did not affect mortality after adjustments for sociodemographic characteristics and health status at the baseline.

Blakely et al^45^	New Zealand	Community	Baseline: 1996 Follow-up: until 1999	Community-dwelling residents aged 25–74 (4.75 million person years) in 1683 census area units (neighborhood area) in 73 regions	Unpaid voluntary activities outside the respondent’s home over 4 weeks (4 items) at neighborhood level and regional level (aggregated). (No individual-level social capital variable was used.)	Mortality (all-cause, cardiovascular disease, cancer, unintentional injury, and suicide)	Multilevel Poisson regression analysis	There was no significant association of neighborhood- or regional-level social capital with any cause of death after adjustment for sociodemographic characteristics.

Blomgren et al^46^	Finland	Community	Baseline: 1990 Follow-up: until 1999	Community-dwelling males aged 25–64 (*n* = 6 516 066) in 84 functional regions	Family cohesion (proportion of persons living alone, of persons divorced by 1993 who were married in 1990, and of 1-parent families from all families with children) and civic participation (voting turnout) in the regions. (No individual-level social capital variable was used.)	Alcohol-related mortality	Multilevel Poisson regression analysis	Low family cohesion and high voter turnout in the region were associated with alcohol-related mortality, and the independent effects of these remained after adjustments for individual sociodemographic characteristics and area-level characteristics (proportion of unemployment, median household income, Gini coefficient, etc).

Lofors and Sundquist^47^	Sweden	Community	Baseline: 1997 Follow-up: until 1999	Entire Swedish population aged 25–64 (*n* = 4 516 787) in 9120 neighborhood units	Mean voting participation at neighborhood unit-level. (No individual-level social capital variable was used.)	First hospitalization for psychosis or depression	Multilevel logistic regression analysis	Low voter participation in neighborhoods was associated with hospitalization for psychosis in both men and women, but not with hospitalization for depression, after adjustment for individual sociodemographic characteristics and neighborhood-level deprivation.

Mohan et al^48^	United Kingdom	Community	Baseline: 1984–1985 Follow-up: until 2001	Community-dwelling adults (*n* = 7578) in 9667 small administrative area units (ie, electoral wards and neighborhood areas)	Engagement in activities (5 items), voting in the last election (1 item), sense of community (5 items), social network (2 items): the proportions of these in the area were used as real indicators of social capital. (No individual-level social capital variable was used.)	All-cause mortality	Multilevel logistic regression analysis	Lower proportions of engagement in activities in the neighborhood area were associated with mortality, but the others did not produce conclusive contextual associations with mortality, after adjustment for age, sex, and health-related behaviors.

Sundquist et al^49^	Sweden	Community	Baseline: 1997 Follow-up: until 1999	Community-dwelling residents aged 45–74 (*n* = 2 805 679) in 9667 small administrative area units (neighborhood areas)	The proportion of people in the neighborhood who voted in the 1998 local government elections as neighborhood-level linking social capital. (No individual-level social capital variable was used.)	First hospitalization for a fatal or nonfatal coronary heart disease (CHD) event	Multilevel logistic regression analysis	Low linking social capital was associated with hospitalization for CHD in both men and women, after adjustment for sociodemographic characteristics.
Islam et al^50^	Sweden	Community	Baseline: 1980–1997 Follow-up: until 2000	Community-dwelling residents aged 20–84 at the time of the interview (*n* = 94 537) in 275 municipalities	Election participation rate and registered number of crimes per 1000 populations as municipal-level social capital. (No individual-level social capital variable was directly defined, but education, age, income, cohabitation status, and number of children in household were used as proxies of individual social capital.)	Mortality (all-cause, cancer, cardiovascular, other diseases, suicide, and other external)	Cox proportional hazards model	Both high election participation rates and low crime rates were protectively associated with individual risk from all-cause mortality for males, particularly among those aged 65+, after adjustment for sociodemographic characteristics and quality of life at the baseline. These associations were not found for females. A high election participation rate and a low crime rate also had protective associations on mortality risks from cancer for males and females aged 65+.

Desai et al^51^	United States	Community	Baseline: 1994–1998 Follow-up: until 1999	Psychiatric patients (*n* = 121 933) discharged from 128 US Department of Veterans Affairs hospitals throughout the United States	Social cohesiveness and trust at state level. (No individual-level social capital variable was used.)	Suicide mortality	Multilevel Poisson regression analysis	Suicide risk was lower in states that had higher social capital, after adjustment for individual sociodemographic characteristics and clinical characteristics.

Kouvonen et al^30^	Finland	Workplace	Baseline: 2000–2002 Follow-up: 2004–2005	Finnish public sector employees (*n* = 33 577; nondepressed respondents at baseline) in 3236 functional work units	Cognitive and structural components of workplace social capital (8 items: sense of cohesion, mutual acceptance, trust for the supervisor, etc) at individual level and work unit level (aggregated).	Self-reported, physician-diagnosed depression; antidepressant treatment	Multilevel logistic regression analysis	Lower individual social capital at work, but not aggregate-level social capital, was associated with subsequent self-reported depression after adjustment for sociodemographic characteristics. No association of individual- or work unit-level social capital with antidepressant treatment was found.

Kouvonen et al^31^	Finland	Workplace	Baseline: 2000–2002 Follow-up: 2004–2005	Finnish public sector employees (*n* = 4853; smokers at baseline) in 1946 functional work units	Cognitive and structural components of workplace social capital (8 items: sense of cohesion, mutual acceptance, trust for the supervisor, etc) at individual level and work unit level (aggregated).	Smoking cessation	Multilevel logistic regression analysis	High individual-level social capital in workplaces was associated with increased likelihood of smoking cessation after adjustment for sociodemographic characteristics, health-related behaviors, and depression. This association was strong in groups with high socioeconomic status (non-manual laborers). Work unit-level social capital was not associated with smoking cessation.

Oksanen et al^32^	Finland	Workplace	Baseline: 2000–2001 Follow-up: 2004	Finnish public sector employees (*n* = 9524; healthy employees at baseline) in 1522 work units	Cognitive and structural components of workplace social capital (8 items: sense of cohesion, mutual acceptance, trust for the supervisor, etc) at individual-level and work unit-level (aggregated).	Self-rated health	Multilevel logistic regression analysis	Both a constantly low level of social capital and a decline in social capital at an individual level were associated with impairment of self-rated health after adjustments for sociodemographic characteristics and health-related behaviors. A constant low level of social capital in work units was marginally associated with risks of poor health, after adjustment for work-unit characteristics.

Väänänen et al^52^	Finland	Workplace	Baseline: 2000–2002 Follow-up: 2004–2005	Finnish public sector employees (*n* = 31 373) in 29 676 work units	Cognitive and structural components of workplace social capital (8 items: sense of cohesion, mutual acceptance, trust for the supervisor, etc) at individual level and work unit level (aggregated).	Co-occurrence of lifestyle risk factors (current smoking, heavy drinking, overweight, and physical inactivity)	Multilevel logistic regression analysis	Social capital at the individual and work unit levels at the baseline was not associated with an increased risk of co-occurrence of lifestyle risk factors at follow-up, after adjustment for sociodemographic characteristics and co-occurrence at baseline.

Approximately half of the studies measured social capital by aggregating survey responses to the area level or workplace level,^23^^,^^24^^,^^30^^–^^32^^,^^45^^,^^52^ while the others used proxy variables derived from administrative databases to measure social capital.^46^^–^^51^ For those that used aggregated variables as area-level or workplace-level social capital, trust and participation in voluntary activities (civic participation) at the individual level were often aggregated to derive measures of area-level or workplace-level social capital. In studies that used existing area-level statistics as social capital variables, voter turnout in the analytic unit was often used. Five articles used individual-level social capital variables in the analysis (1 article in a community setting^23^ and 4 in a workplace setting^30^^–^^32^^,^^52^), while the other studies did not include individual-level variables of social capital. Regarding outcome variables, mortality was set as the outcome in 6 articles^24^^,^^45^^,^^46^^,^^48^^,^^50^^,^^51^; hospitalization,^47^^,^^49^ self-rated health,^23^^,^^32^ and health-related behavior were used as outcomes in 2 articles each^31^^,^^52^; and depression was used as the outcome in 1 article.^30^

### Mortality

Studies of all-cause mortality reported both positive and negative contextual effects of social capital. Mohan et al reported that less engagement in neighborhood activity lowered all-cause mortality,^48^ and Islam et al found a limited protective effect of municipal-level social capital on mortality among men, with a particularly strong effect among those aged 65 years or older.^50^ In contrast, another study found that the density of community social networks had a detrimental effect on mortality, although community collective efficacy had a protective association.^24^ In a study in New Zealand, Blakely et al found no association between neighborhood social capital and all-cause mortality.^45^

Regarding cause of death, 1 study reported a protective contextual effect of social capital on suicide,^51^ but 2 other studies concluded that social capital had no contextual effects on suicide.^45^^,^^50^ Only 1 study of alcohol-related mortality found a protective effect for regional-level social capital.^46^ Two studies examined cancer-related mortality as an outcome: 1 reported that high municipal-level social capital had a limited protective effect against cancer-related mortality among adults aged 65 years or older,^50^ and the other showed no association between neighborhood social capital and cancer-related mortality.^45^

### Hospitalization

Hospitalizations for coronary heart disease (CHD), psychosis, and depression were examined as outcome variables in 2 studies. The contextual protective effects of social capital were demonstrated in hospitalizations for CHD and psychosis,^47^^,^^49^ but no association was found for hospitalizations due to depression.^47^

### Self-rated health

A study in a community setting found that both high individual- and area-level social capital (trust) were inversely associated with poor self-rated health.^23^ In a workplace setting, Oksanen et al reported that lower levels of social capital, at both individual- and workplace-levels, were associated with poor self-rated health.^32^

### Health-related behavior

Two studies of social capital and health-related behavior have been conducted in workplace settings. One focused on smoking cessation as the outcome, and the other examined co-occurrence of lifestyle risk factors. Kouvonen et al reported that high individual social capital, but not workplace social capital, was associated with smoking cessation.^31^ Väänänen et al found no association between both individual- and workplace-level social capital and the co-occurrence of lifestyle risk factors.^52^

### Depression

An association between lower individual-level social capital and self-reported, physician-diagnosed depression was found among Finnish employees, but there was no association between workplace-level social capital and depression.^30^

## DISCUSSION

We identified 13 published studies of social capital and health that used prospective data and multilevel analysis. These studies were mainly conducted in Western countries. North American studies have tended to focus on community settings, while Scandinavian studies contribute to the empirical literature on workplace social capital. Multilevel evidence from Asian settings was limited. We identified at least 10 cross-sectional multilevel studies of social capital and health on community, workplace, and school settings in Japan.^16^^,^^19^^,^^20^^,^^22^^,^^25^^–^^29^^,^^33^ However, there have been no prospective studies from Asian countries.

Our review indicates that both area/workplace social capital and individual social capital generally appear to have positive effects on health outcomes, although the studies varied with regard to participants, setting (including country), follow-up period, and variables used as social capital and health outcomes. Due to the limited number of studies, the robustness of the evidence is questionable. In the 13 reviewed articles, the cognitive and structural dimensions of social capital were analyzed separately in some studies and combined in others. Social capital does not always generate a beneficial effect on health outcomes: the effect of social capital might provide a benefit for 1 population while disadvantaging another.^53^^,^^54^ It is expected that further research will identify dimensions of social capital that positively or negatively affect health outcomes. One direction for future research was suggested by a recent Japanese study of individual-level access to bonding and bridging social capital.^55^ In that cross-sectional study, higher bridging social capital (as assessed by respondents to a survey who reported that they participated in civic groups that were heterogeneous with respect to age group, gender, and occupation) was strongly associated with higher self-rated health. By contrast, bonding social capital was not significantly associated with self-rated health. The association of bridging capital with health was more pronounced in women than in men. To corroborate these findings, additional research in a longitudinal, multilevel framework is needed.

Unfortunately, we were not able to find an empirical intervention study that documented a health improvement resulting from increased social capital in the community. There is no easy way to build social capital. Fostering social capital requires significant material and human resources. Prevention and intervention efforts have traditionally targeted either the general population (through, for instance, the mass media) or individuals who are at risk for adverse health outcomes. There have been some prospective multilevel studies conducted in communities.^23^^,^^24^^,^^45^^–^^51^ The results of these studies support associations between social capital at a neighborhood-level (or geographic area) and different aspects of health outcomes, which implies that neighborhoods or other social contexts with low contextual levels of social capital should be targeted.

**Figure 2.**
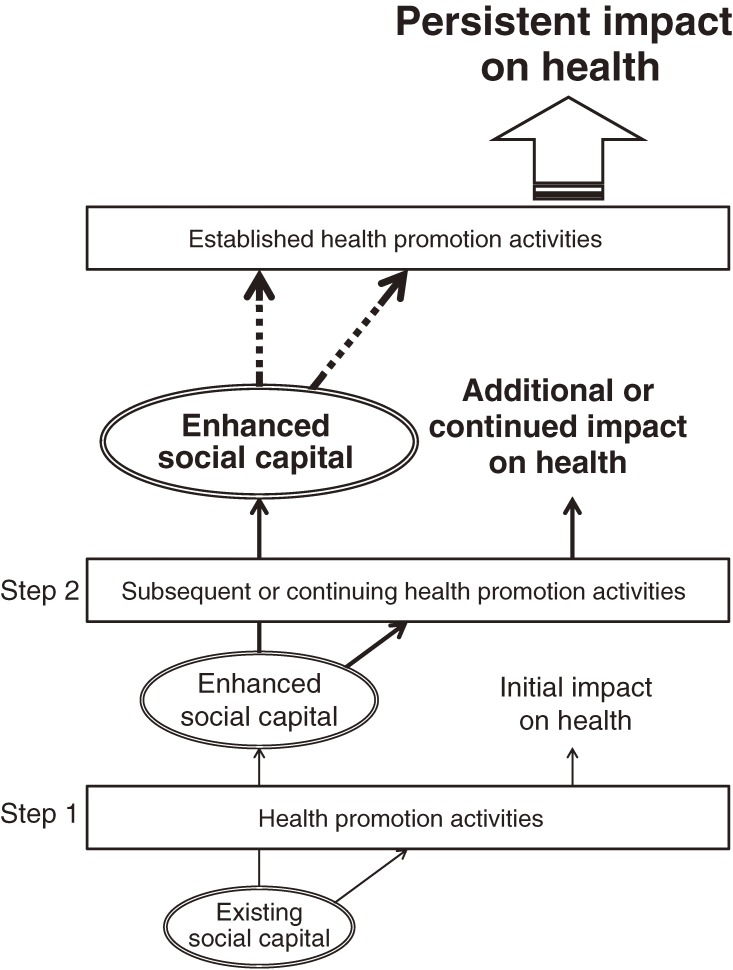
Image of desired relationship between social capital and health promotion intervention programs

Social capital does not incidentally arise in communities. Rather, it is itself shaped by the broader structural forces operating at the community-level, such as historical patterns of residential mobility and municipal investment in housing and local infrastructure, as well as policies that perpetuate residential segregation or planned reductions in services and amenities.^12^ Moreover, the building of social capital must be considered as a complement to, rather than a replacement for, broader structural interventions.^7^ Figure 2 shows the relationship between social capital in the community and health promotion activities (intervention programs). Every community has their own level and type of social capital. The existing social capital within a community—which is closely related to civic mobilization, sense of coherence, and commitment—can influence both the efficiency and effectiveness of a program. Therefore, the health effectiveness of a program may depend on not only the program itself and the individual participants, but also on community social capital. At the same time, social capital can be affected (preferably enhanced) by the implementation of a program. Enhanced social capital can influence the next program or continuation of the current program, as well as the effect of the program on the community. This cycle enables the program to have a continuing effect on health in the community. Thus, intervention programs and social capital have a reciprocal relationship.

The Experience Corps is a social approach to health promotion that was initially conducted in the American city of Baltimore, Maryland and used elderly volunteers in the community.^56^ The program places a critical mass of older adult volunteers in public elementary schools to generate a substantial individual-level impact on the educational outcomes of children and improve the health and well-being of the volunteers.^56^^,^^57^ The Experience Corps uses public elementary schools as the core of the intervention program. It was designed to have an impact on school-level and community-level social capital as well as individual-level social capital and involved children, their parents, teachers, and residents in the community to encourage multilevel interactions (individual-, school-, and community-level).^57^^,^^58^

Health promotion interventions that target only individual behavior have a less-than-expected impact on health outcomes. If the intervention is to be conducted in the community and is intended to target community residents, then the broader social context must be considered.^59^

### Conclusion

In future research on social capital, both multilevel approaches and prospective data analysis will be important in accumulating robust evidence and developing intervention strategies that rely on social capital in the community (including the workplace) for health promotion. Studies need to clarify the specific dimensions or forms of social capital that would be most effective in interventions. In addition, they must identify the health outcomes that would be improved by increasing social capital and the beneficiaries of such improvements. There have been few multilevel prospective studies of social capital and health in Asia. However, the rapidly aging population in Asia, especially in Japan, and China’s explosive population growth are of global concern, and the reconsideration and rebuilding of communities in Asian countries will become a significant issue. Regrettably, an effective intervention strategy to build social capital has yet to be devised, possibly due to the multiplicity of definitions and the diverse dimensions of social capital. These factors make it difficult to develop and evaluate intervention strategies. As indicated by the present literature review, prospective epidemiologic evidence on the effect of social capital on health is very limited. To effectively translate the epidemiologic findings on the association between social capital and health to practice, we must demonstrate the feasibility of building social capital in its various forms.
